# Curcumin inhibits cancer stem cell phenotypes in *ex vivo* models of colorectal liver metastases, and is clinically safe and tolerable in combination with FOLFOX chemotherapy

**DOI:** 10.1016/j.canlet.2015.05.005

**Published:** 2015-08-10

**Authors:** Mark I. James, Chinenye Iwuji, Glen Irving, Ankur Karmokar, Jennifer A. Higgins, Nicola Griffin-Teal, Anne Thomas, Peter Greaves, Hong Cai, Samita R. Patel, Bruno Morgan, Ashley Dennison, Matthew Metcalfe, Giuseppe Garcea, David M. Lloyd, David P. Berry, William P. Steward, Lynne M. Howells, Karen Brown

**Affiliations:** a Department of Cancer Studies, University of Leicester, Leicester Royal Infirmary, Leicester, LE2 7LX, UK; b Department of Hepatobiliary Surgery, Leicester General Hospital, Gwendolen Road, Leicester, UK; c Department of Hepatobiliary Surgery, University Hospitals of Wales, Cardiff, UK

**Keywords:** Colorectal liver metastases, Curcumin, Cancer stem cells, Combination therapy

## Abstract

*In vitro* and pre-clinical studies have suggested that addition of the diet-derived agent curcumin may provide a suitable adjunct to enhance efficacy of chemotherapy in models of colorectal cancer. However, the majority of evidence for this currently derives from established cell lines.

Here, we utilised patient-derived colorectal liver metastases (CRLM) to assess whether curcumin may provide added benefit over 5-fluorouracil (5-FU) and oxaliplatin (FOLFOX) in cancer stem cell (CSC) models. Combination of curcumin with FOLFOX chemotherapy was then assessed clinically in a phase I dose escalation study. Curcumin alone and in combination significantly reduced spheroid number in CRLM CSC models, and decreased the number of cells with high aldehyde dehydrogenase activity (ALDH^high^/CD133^−^). Addition of curcumin to oxaliplatin/5-FU enhanced anti-proliferative and pro-apoptotic effects in a proportion of patient-derived explants, whilst reducing expression of stem cell-associated markers ALDH and CD133. The phase I dose escalation study revealed curcumin to be a safe and tolerable adjunct to FOLFOX chemotherapy in patients with CRLM (n = 12) at doses up to 2 grams daily.

Curcumin may provide added benefit in subsets of patients when administered with FOLFOX, and is a well-tolerated chemotherapy adjunct.

## Highlights

• Curcumin + FOLFOX inhibits growth of primary cancer stem cell (CSC) spheroid models.
• Curcumin + FOLFOX decreases expression of CSC markers in primary CSC spheroid models.
• Curcumin enhances proapoptotic effects of chemotherapy in explant culture.
• Curcumin is safe and tolerable in combination with FOLFOX chemotherapy.
• Curcumin is perceived by patients as an acceptable daily adjunct to chemotherapy.

## Introduction

Twenty-five percent of patients diagnosed with colorectal cancer present with metastatic disease [1], with the liver being the predominant site for metastatic spread. Approximately 50% of colorectal cancer (CRC) patients have liver metastases either at primary diagnosis or following disease recurrence [2]. Surgical resection, where possible, is the only potentially curative option in locally recurrent disease or metastatic disease localised to the liver or lungs. However, the majority of patients are not suitable for surgery and receive palliative treatment using multi-agent chemotherapy with or without biological agents.

Addition of experimental agents into standard care chemotherapy (5-FU, oxaliplatin, folinic acid (FOLFOX)) is often accompanied by an increase in debilitating side effects. Oxaliplatin-based chemotherapy results in acute and chronic peripheral neuropathies which are frequently dose-limiting, leading to early treatment cessation or treatment de-escalation protocols. Consequently, treatment efficacy is likely to be compromised. Increasingly, pre-clinical evidence suggests that there may be a role for low-toxicity, dietary-derived agents in enhancing efficacy or reducing chemotherapy-induced side effects. One such agent which has been investigated extensively for chemopreventive and therapeutic effects in colorectal cancer is the polyphenol, curcumin, a constituent of the spice turmeric [3–6]. Clinical trials utilising curcumin in an oncology setting have targeted several disease sites including colorectal [5,7–11], pancreatic [12–15], breast [16], and haematological malignancies [17,18], in addition to being investigated for its ability to alleviate therapy-induced toxicities such as radiation dermatitis [19,20]. Whilst these are generally small, phase I/II trials, the favourable toxicity profile of curcumin combined with limited evidence that certain subsets of patients may derive benefit from its addition to interventional regimens warrants further investigation. These studies have also alluded to pharmacodynamic changes which may bear relevance to the carcinogenic process. In colorectal cancer, this includes decreased leukocyte prostaglandin E2 levels [11], reduced levels of the oxidative DNA adduct M_1_G in malignant tissue [8], reduction in the number of aberrant crypt foci [7], and up-regulation of p53 in tumour tissue [10]. In pancreatic cancer, curcumin was found to downregulate Nuclear Factor kappa B (NFκB), cyclo-oxygenase 2(COX-2) and phosphorylation of Signal Transducer and Activator of Transcription 3 (STAT-3) in peripheral blood mononuclear cells [12], whilst a decrease in nitric oxide was observed in chronic myeloid leukaemia patients [17].

Further to these published studies, there are currently 13 actively recruiting oncology-based trials using curcumin as an investigational agent registered on clinicaltrials.gov.

More recent evidence suggests that curcumin may be able to target cancer stem-like cells (CSCs) [21–23]. It has been postulated that the CSC component of tumours contributes to decreased efficacy of chemotherapy, and may ultimately be responsible for tumour recurrence and metastatic spread. These cells have the capacity for self-renewal, differentiation, invasion and metastasis, and are thought to have a higher degree of innate or acquired chemo-resistance [24,25]. Thus failure to eradicate these cells may ultimately be responsible for disease progression.

In the study described here, *ex-vivo* models derived from human colorectal liver metastases were utilised to test the hypothesis that addition of curcumin to FOLFOX chemotherapy enhances efficacy, with the potential to target cancer stem-like cells. Despite growing evidence suggesting curcumin may be able to augment FOLFOX chemotherapy, this combination has never been tested clinically. Therefore, here we also describe, for the first time, a dose escalation study combining curcumin with first line FOLFOX chemotherapy in patients with CRLM.

## Materials and methods

### Laboratory methods

#### Collection and processing of tumour samples

Tumour samples were obtained from patients undergoing surgical resection for colorectal liver metastases at University Hospitals of Leicester NHS Trust. Ethical approval for this fully anonymised, excess tissue study was granted by Leicestershire, Northamptonshire and Rutland Ethics Committee (REC reference 09/H0402/45).

Tissues were processed as previously described [26]. Stem-like and epithelial components were characterised by flow cytometry (FACS Aria II, Becton Dickinson, Oxford, UK) using antibodies directed against epithelial cellular adhesion molecule (EPCAM), CD133, CD26 and aldehyde dehydrogenase activity.

#### Maintenance of tumours and sphere growth

Tumour tissues were maintained by serial passage through Non Obese Diabetic Severe Combined Immunodeficient Mice (NOD-SCID) mice [26]. All experiments were carried out under project license PPL 80/2167, granted to Leicester University by the United Kingdom Home Office, vetted by Leicester University Local Ethical Committee for Animal Experimentation and met the standards required by the UKCCR for animal welfare. Following serial passage, part of the tumour tissue was collagenase digested for flow cytometric profiling and assessment of spheroid formation, and a portion was formalin-fixed and paraffin wax-embedded. Spheroids were treated with pharmacologically relevant concentrations of agents as follows: DMSO, 5 µM curcumin, 2 µM oxaliplatin + 5 µM 5-FU and 5 µM curcumin + 2 µM oxaliplatin + 5 µM 5-FU. Following a period of 2 weeks (representing 1 cycle of chemotherapy), spheroids were harvested for FACS analysis, spheroid counts and size using a Nikon TE 2000 U camera system with Eclipse software (Surrey, UK). A pluripotent stem cell protein profiler array kit (R&D Systems, Oxfordshire, UK) was used to further determine treatment-induced effects on the stem cell compartment.

#### Explant culture

Six-well plates and inserts (0.4 µm, Millipore, Watford, UK) were equilibrated at 37 °C, 5% CO_2,_ with 1.5 mL of media/well (DMEM, 1% FCS and antibiotic/antimycotic). Tumour tissue was cut (1 mm [3]), 9 segments randomly allocated per well, placed on top of the inserts and incubated for 15 h. Media containing appropriate treatments were added and tissues incubated for a further 24 h prior to formalin fixation/paraffin embedding. Immunohistochemistry was undertaken using the Novolink Polymer Detection as per manufacturer's instructions, and the following markers were assessed: Ki67, cleaved caspase-3 (quantitative assessment), ALDH1A1, CD133, CD26, Nanog, XRCC1, ERCC1, MLH1, MSH2, MSH6 (semi-quantitative assessment via a trained pathologist). Scoring was undertaken blinded to the treatment groups.

### Clinical methods – phase I dose escalation study to assess safety, tolerability and feasibility of administering oral curcumin during standard FOLFOX chemotherapy for palliation of colorectal liver metastases (ClinicalTrials.gov:NCT01490996)

Ethical approval was obtained from the Nottingham Research Ethics Committee 1 (REC reference 11/EM/0263). Target populations were patients presenting with histological diagnosis of metastatic colorectal cancer suitable for palliative FOLFOX-based chemotherapy. The Trial schema is shown in Supplemental data S1, and full details of the trial protocol in accordance with the SPIRIT statement [27] can be found in Ref. 28. In brief, the first tier of participants was recruited to receive 500 mg (1 capsule) of oral curcumin C3-complex daily, 7 days prior to the scheduled chemotherapy. If no curcumin-induced toxicities (CIT) were observed, daily oral curcumin was continued once chemotherapy commenced. FOLFOX-based chemotherapy consisted of 2-weekly cycles of chemotherapy given to a maximum of 12 cycles or until withdrawal from the trial. Once no CITs were observed in the first tier of patients following completion of two cycles of chemotherapy, recruitment to the next escalation dose of 1 g (2 capsules) daily and then 2 g (4 capsules) commenced. Side effects were classified using the Common Terminology Criteria for Adverse Events (CTC-AE) version 4, with further data captured using a validated neurotoxicity questionnaire and pre/post trial questionnaires to capture demographics and concerns regarding curcumin consumption.

## Results

### Effect of curcumin combined with 5-FU + oxaliplatin in spheroid models of CRLM

**Fig. 1:**
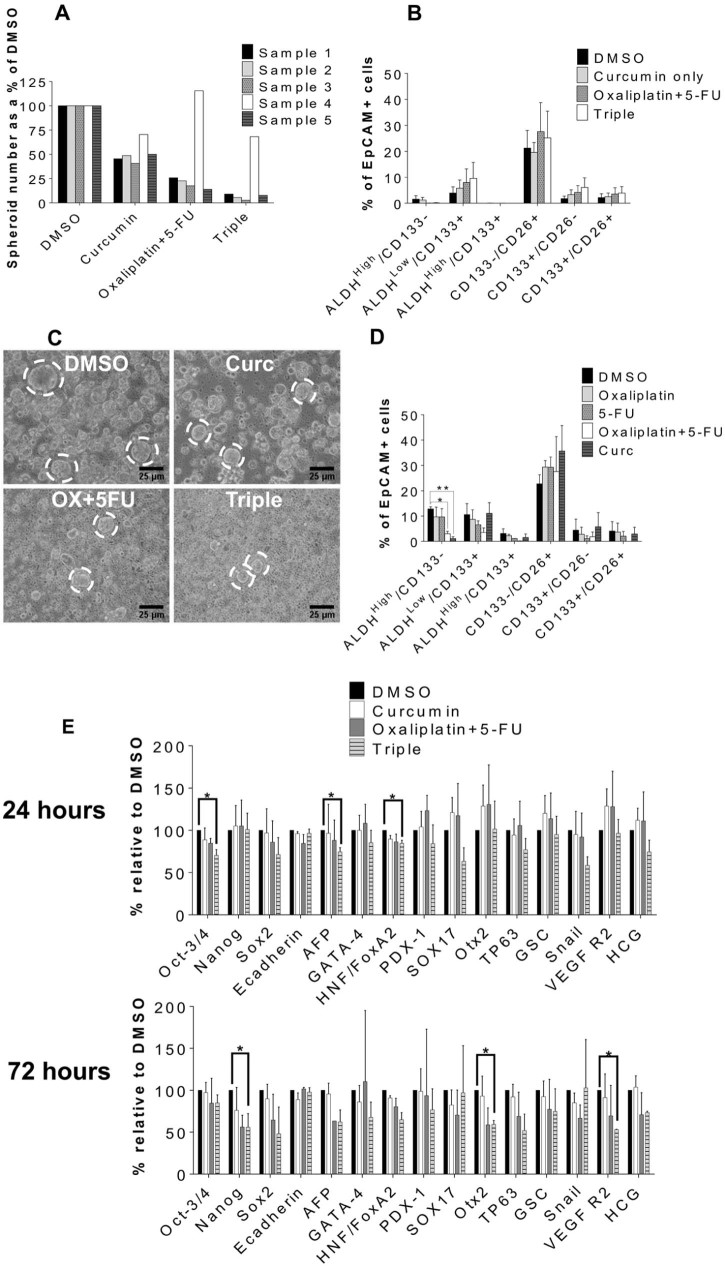
The effects of combinations of curcumin, oxaliplatin and 5-FU on spheroids and stem cell markers, from single cells derived from serial passage of CRLM tissue in NOD-SCID mice (A–C, E) or directly from CRLM without passage (D). (A) Spheroid number after treatment, represented as a percentage of the DMSO control, *N* = 5 (each replicate is a different patient sample). (B) Flow cytometric data as a percentage of the EpCAM+ population in spheroids from (A) ±SEM, *N* = 5. (C) Representative light microscopy of spheroids after treatment. Circles highlight examples of spheroids. (D) Flow cytometry of spheres derived from 1 patient sample over 3 passages. (E) Spheroids were treated for 24 or 72 hours. Expression is represented as a percentage of DMSO as determined by densitometry. Mean ± SEM, *N* = 3, * *P* ≤ 0.05, ** *P* ≤ 0.001 compared to DMSO.

Spheroids derived from five different patient CRLM xenografts were incubated for 2 weeks with either oxaliplatin + 5-FU alone or in combination with curcumin. Treatments decreased spheroid numbers compared to control (Fig. 1A,C), but significance was only reached with the triple combination, reducing spheroid number by ~80% (Supplemental data S2). The ALDH^high^/CD133^−^ population was obliterated by oxaliplatin + 5-FU alone and by the triple combination (Fig. 1B), but there were no significant differences in the populations. In one sample (in which high ALDH activity was associated with aggressive spheroid growth without prior passage), curcumin alone decreased the ALDH^high^/CD133^−^ population to a greater extent than oxaliplatin + 5-FU (Fig. 1D). Exposure of spheroids to the triple combination significantly downregulated expression of pluripotent stem cell markers Oct3-4, AFP and HNF/FoxA2 at 24 hours, and Nanog, Otx2 and VEGFR2 at 72 hours (Fig. 1E).

### Effect of curcumin combined with 5-FU + oxaliplatin on stem cell markers in explant cultures of CRLM

Explant cultures of patient-derived tissues were exposed to oxaliplatin + FU alone or in combination with curcumin. Stem cell markers were assessed immunohistochemically, and staining intensity graded in comparison with cultures treated with either DMSO or oxaliplatin + 5-FU. ALDH1A1 staining was decreased by curcumin alone and oxaliplatin + 5-FU in 4/10 or 2/10 explants, respectively (Table 1). In cultures exposed to curcumin + oxaliplatin expression of ALDH1A1 or CD133 was inhibited in 3/10 or 4/9 samples, respectively, compared to staining in tissues exposed to oxaliplatin + 5-FU. Expression of both CD26 and Nanog remained relatively refractory to treatment, with only the triple combination decreasing CD26 in 2/10 samples.

### Effect of curcumin on Ki67 and cleaved caspase-3 immunoreactivity in explant cultures of CRLM

**Fig. 2:**
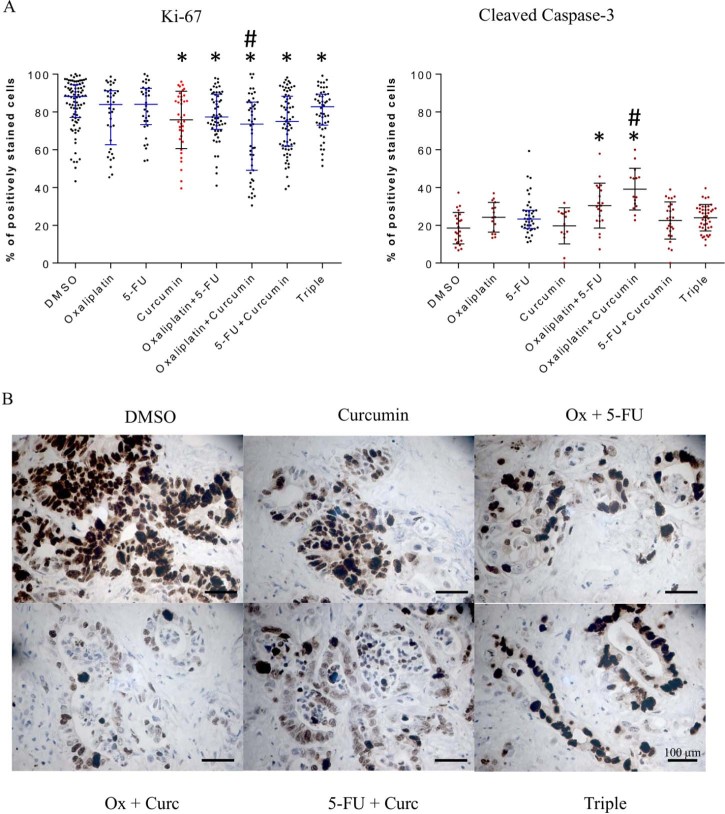
Example ki67 and cleaved caspase-3 scoring of explant immunostaining (A) and sample explant micrographs immunostained for ki67 (B). (A) Normally distributed data are displayed as red dots, black error bars, the average and the standard deviation. Non-normally distributed data are displayed as black dots with blue error bars. Significant differences are indicated where * represents P ≤ 0.05 compared to DMSO, # corresponds to P ≤ 0.05 compared to OX + 5-FU. Each data point represents an average score from one field of view. Scoring was undertaken by 2 independent observers, and the scores averaged. (B) Sample ki67 explant micrographs shown at ×40 objective. (For interpretation of the references to colour in this figure legend, the reader is referred to the web version of this article.)

Curcumin alone significantly decreased proliferation, as reflected by Ki67 staining, and increased apoptosis in 3/8 and 5/8 explants, respectively, when compared to DMSO (sample staining and quantification shown in Fig. 2). The combination of oxaliplatin + curcumin appeared most efficacious, significantly decreasing proliferative index in 6/8 and inducing apoptosis in 8/8 explants (Table 1).

No treatment-induced changes to XRCC1, ERCC1, MLH1, MSH2 or MSH6 were observed in any samples (Supplemental data S3).

### Design of clinical study

The phase I dose escalation trial was designed to comprise three tiers differing in daily curcumin dose (0.5, 1 or 2 g). Three participants recruited to tier 1 completed 2 cycles of chemotherapy without curcumin-related toxicity, fulfilling the criteria for recruitment to the second tier. Likewise, dose-escalation to the third tier began after 3 patients from tier 2 had completed 2 cycles of chemotherapy without curcumin-induced toxicities. One patient from the initial three recruited into tier 3 experienced diarrhoea, which was deemed to be related to curcumin. Therefore three further patients (6 in total) were entered into, and completed tier 3 (2 g curcumin daily). Fifty percent of patients completed 12 cycles of chemotherapy with curcumin. The average compliance rate (as determined by returned curcumin capsules) across all participants was 93.8%. Comparing the 3 tiers, mean compliance rates were 84, 100 and 96% for tiers 1, 2 and 3, respectively. Five patients (41.7%) were 100% compliant.

### Clinical effects of curcumin alone or in combination with FOLFOX chemotherapy

*Side effects:* Initially curcumin was administered as a single agent for one week prior to commencing chemotherapy. Five of twelve participants (41.7%) experienced no curcumin-induced adverse events (AE) during the week pre-chemotherapy. The most common curcumin-induced side effects were constipation (25.0%), dry mouth (16.7%) and flatulence (16.7%). All AEs reported were grade 2 or less and did not prevent patients from progressing to chemotherapy. Once participants received chemotherapy in combination with curcumin, the most commonly reported side effect was peripheral neuropathy, noted in eleven patients (91.7%). Other toxicities were fatigue (83.3%), diarrhoea (66.7%) and oral mucositis (58.3%). AEs possibly attributable to curcumin, but which are also common side effects of chemotherapy, are shown in Table 2, and appeared unrelated to curcumin dose (grade 3 AEs as percent total for Tier 1 = 16%; Tier 2 = 6%; Tier 3 = 7% total). The majority of AEs (90.0%) were classed as grade 1 or 2. Three patients described grade 3 toxicities that could have been attributed to curcumin. Grade 3 diarrhoea was deemed dose-limiting for one patient, therefore the curcumin dose was de-escalated from 2 grams, and maintained at 1 gram. Other grade 3 toxicities, likely unrelated to curcumin, included hyponatraemia, neutropenia, thromboembolic events, urinary tract infections and weight loss. One grade 5 event occurred where a patient was admitted with a severe chest infection and subsequently died. This event was not deemed to be due to curcumin.

### Disease response

Eleven of twelve participants (91.7%) showed stable disease or partial response to treatment after 6 cycles of chemotherapy. A reduction in target lesions of at least 30% (partial response) was recorded in more than half of patients (58.3%), with the best response being a 75.8% reduction. At the end of treatment, eight of the remaining ten participants (80%) maintained a partial response or stable disease. Median progression-free survival was 34 weeks. EORTC QLQ30 global health and functionality scores suggested worsening treatment-induced symptoms, but increased functionality post trial compared with pre-trial scores (Supplemental data S4).

### Acceptability of curcumin

It was important to assess patients' perceptions of curcumin, if it is to be used as a chemotherapy adjunct in future trials. Most patients (91.7%) were ‘not at all’ concerned about the once daily dosing frequency. By treatment end, the majority of patients (72.7%) reported no difficulties with capsule number, size or frequency of their curcumin dose. Potential side-effects of treatment were a minor concern for 41.7% of patients prior to commencing treatment. By treatment end, 81.8% had no concerns regarding side-effects from curcumin (Supplemental data S5).

## Discussion

Curcumin has been extensively investigated for potential chemopreventive or anti-tumour effects in colorectal cancer. Much of the research has classically focussed on adherent cell lines, or use of animal models exhibiting APC loss [4,29–32]. The discovery of novel anticancer therapies now preferentially involves the use of models which have greater resemblance to clinical disease [33–35], primarily by utilising tissues resected from patients. This allows study of drug action across the heterogenous phenotypic/genotypic cohorts representative of the malignancy under study. CRLM models have thus far rarely been used to study the efficacy of chemotherapy agents in colorectal cancer. Cancer stem cells are increasingly proffered as a target to prevent disease recurrence or to circumvent therapeutic resistance. Therefore, regimens which may be able to target both the rapidly proliferating differentiated cells concurrent with less frequently dividing stem-like cells may improve overall therapeutic efficacy. We and others have utilised the established colorectal cancer cell lines HCT-116 and HT-29 to assess potential for the curcumin/oxaliplatin/5-FU combinations to down-regulate the stem-like phenotype [4,36–39]. Curcumin alone and in combination with chemotherapy was observed to inhibit spheroid formation and downregulate stem cell associated markers CD44 and CD166 and ALDH activity, in addition to downregulation of epidermal growth factor, insulin-like growth factor and Notch, all of which may have involvement in maintenance of the stem-like phenotype. Following on from this data, it was essential to translate these findings to models with greater relevance to colorectal cancer patients. The findings presented here suggest for the first time that curcumin may enhance oxaliplatin/5-FU-based chemotherapy in models derived directly from patients for whom the treatments are ultimately intended. The observed reduction of spheroid number by the combination of 5-FU/oxaliplatin  +curcumin is indicative of the ability of this combination to affect the cancer stem cell population. In addition, curcumin alone decreased spheroid number to a greater extent than the 5-FU/oxaliplatin treatments. Previously we have shown that spheroid forming capacity in CRLM is reflected by the order of expression of ALDH^high^ > CD133 > CD26 [26]. This order suggests that reduction in ALDH activity correlates with reduced spheroids and fewer CSCs [26]. Transcription factors associated with the ALDH^high^ stem-like phenotype (Oct 3/4, Nanog, Otx2, AFP) were also downregulated by the combination of 5-FU/oxaliplatin + curcumin. Explant cultures corroborated that combinations including curcumin were able to decrease CSC markers when compared to oxaliplatin + 5-FU for ALDH, CD133 and Nanog. Differential treatment responses were not due to donor differences in expression of proteins associated with 5-FU resistance (XRCC1, ERCC, MLH1, MSH2 or MSH6) [40,41] (Supplemental data S3).

There has been much speculation as to whether curcumin may prove a suitable adjunct to chemotherapy in clinical regimens, and here we established that combination of curcumin with FOLFOX chemotherapy in CRLM patients was safe and tolerable in the first line setting. Patients generally tolerated their week of curcumin (pre-chemotherapy) with minimal AEs. The events reported were primarily gastrointestinal and consistent with those described in previous curcumin trials [3]. Side-effects were mild (Grade 1 or 2), either resolving spontaneously or following medication. Addition of curcumin to FOLFOX was well tolerated, with a compliance rate of 93.8%. Increasing curcumin dose did not affect compliance, which was in keeping with other oral antineoplastic agents such as Tamoxifen and Capecitabine (median compliance rates of 72–96%, 86.7–100% respectively) [42,43]. Diarrhoea was the main serious adverse event possibly associated with curcumin. Diarrhoea is commonly observed in patients treated with the combination of FOLFOX and Bevacizumab (11%) [44], and FOLFOX alone, with grade 3–4 diarrhoea reported in 10% of patients [45]. It was therefore difficult to determine how much each treatment component contributed to the grade 3 diarrhoea observed in two patients here.

Median PFS and treatment response were largely comparable with that previously reported for FOLFOX and FOLFOX + Bevacizumab [46], although it is important to note that the limited patient numbers in this study do not permit conclusions to be drawn regarding potential efficacy. Whilst approximately 55% of trial participants reported deterioration in their therapy-induced symptoms by the end of their treatment, the majority (72.7%) reported that they would be willing to continue curcumin for as long as was necessary. This reflected the perception by participants that most of their side-effects were due to chemotherapy, resulting in favourable opinions on tolerability and feasibility of this treatment.

This study reports for the first time that addition of curcumin to FOLFOX-based chemotherapy enhances efficacy in patient-derived CRLM cultures. Greater pro-apoptotic and CSC targeting efficacy was observed for curcumin than for oxaliplatin and 5-FU in a small patient subset, warranting further investigation to determine factors that influence response to curcumin. For the first time, safety and tolerability for curcumin in combination with FOLFOX chemotherapy are reported. A randomised phase II study comparing participants receiving FOLFOX only with those receiving FOLFOX + curcumin is currently recruiting.

## Funding

This work was supported by two studentships from Hope Against Cancer, Leicester, UK, The Royal College of Surgeons (Eng), The Bowel Disease Research Foundation, by Cancer Research UK on a programme grant [C325/A13101] and by Cancer Research UK in conjunction with the Department of Health on an Experimental Cancer Medicine Centre grant [C325/A15575].

## Authors' contributions

**MIJ**: *Ex vivo* laboratory work, manuscript preparation; **CI**: recruitment and monitoring of patients in phase I, regulatory paperwork, manuscript preparation; **GI**: study design, original regulatory and ethics submissions, obtained funding; **AK**: *ex vivo* laboratory work; **JAH**: flow cytometry; **NG-T**: patient recruitment; **AT**: study design, patient recruitment, manuscript preparation; **PG**: pathology; **HC**: *in vivo* work; **SRP**: sample collection and processing; **BM**: radiology input; **AD, MM, GG, DML**: surgical input, manuscript preparation; **DPB**: surgical input, ethics submission (*ex vivo*), manuscript preparation; **WPS** study design, ethics submission, obtained funding, manuscript preparation; **LMH**: study design, obtained funding, managed research, wrote manuscript; **KB**: study design, obtained funding, manuscript preparation. All authors read and approved the final manuscript.

## Conflict of interest

None to declare.

## Figures and Tables

**Table 1:** Summary of the effects of treatment combinations on TIC and proliferative markers in explant culture.

Treatment observations	TIC marker	Curcumin	Oxaliplatin + curcumin	5-FU + curcumin	Oxaliplatin + 5-FU	Triple
More efficacious than DMSO	ALDH1A1	4/10	4/10	2/10	2/10	3/10
	CD133	3/9	5/9	4/8	3/9	2/7
	CD26	1/10	1/8	1/10	1/10	2/10
	Nanog	1/9	1/9	1/9	1/9	1/9
	Ki67	3/8	6/8	5/8	2/8	4/8
	Cleaved caspase-3	5/8	8/8	5/8	7/8	5/8
More efficacious than oxaliplatin + 5-FU	ALDH1A1	3/10	3/10	1/10	N/A	2/10
	CD133	3/9	4/9	3/8	N/A	1/7
	CD26	0/10	0/8	0/10	N/A	1/10
	Nanog	2/9	2/9	2/9	N/A	1/9
	Ki67	0/8	3/8	1/8	N/A	0/8
	Cleaved caspase-3	2/8	2/8	1/8	N/A	2/8

CD133, ALDH1A1, CD26 and Nanog expressions were assessed following treatment with combinations of curcumin, oxaliplatin and 5-FU. A pathologist, blinded to the treatment groups, assessed expression of TIC markers. Samples were scored using a semi-quantitative +, ++ and +++ grading system based on staining intensity and number of stained cells. Samples were counted as “more efficacious” if it was at least a single ‘+’ sign less than that for the DMSO or FOLFOX groups. For ki67 and cleaved caspase-3, absolute numbers of positively stained cells were counted in each treatment group. All data were tested for normality prior to applying a paired sample T test. For samples exhibiting both normally and non-normally distributed data across treatments, parametric and non-parametric T tests were applied appropriately. N/A = not applicable.

**Table 2:** Treatment side effects.

Adverse event	No. of patients	% of patients	Grade pre-chemotherapy	Grade with chemotherapy	Grade 3 No. of patients	Grade 3 % of events
Abdominal pain	4	33.3%	1,2	1,2,3	1	16.7%
Acute kidney injury	1	8.3%	–	1,3	1	33.3%
Anorexia	4	33.3%	–	2,3	3	75.0%
Bloating	1	8.3%	2	–	–	–
Constipation	5	41.7%	1	1,2	–	–
Diarrhoea	8	66.7%	1	1,2,3	2	30.8%
Dry mouth	3	25.0%	1	1	–	–
Dyspepsia	4	33.3%	1	2	–	–
Flatulence	3	25.0%	1	2	–	–
Oral mucositis	7	58.3%	–	1,2,3	2	16.7%
Nausea	4	33.3%	1	1,2,3	1	16.7%
Rash	2	16.7%	–	1,2	–	–
Vomiting	2	16.7%	–	1,2	–	–
Weight loss	4	33.3%	–	1,2,3	1	20.0%

Side effects may have been attributable to curcumin, when given in combination with FOLFOX chemotherapy.

## Supplementary material

Supplemental data S1 CUFOX Phase I dose escalation trial schema.

Supplemental data S2 Spheroid number after treatment, represented as a percentage of the DMSO control. *N* = 5 (each replicate is a different patient sample). ±SEM, * = *P* ≤ 0.05 compared to DMSO, # = *P* ≤ 0.05 compared to curcumin alone.

Supplemental data S3 Table shows basal expression of DNA mis-match repair proteins by IHC in the samples used for explant culture, and sample staining patterns. A pathologist assessed the staining for each explant and assigned a score of either +, ++ or +++ with more plus symbols representing a greater amount of expression between samples. No changes to staining patterns were observed following any treatment.

Supplemental data S4 Table shows Global health score and functionality for all participants as assessed using EORTC QLQ30. C1 001, 002, 003 = tier 1, C1004, 006, 007 = tier 2, C1 008, 009, 010, 012, 013 = tier 3. C1 011 for tier 3 = RIP so no post-trial data were obtained.

Supplemental data S5 Results of curcumin questionnaires showing responses pre- and post-trial.
