# Supplementary figures and images for: Curcumin inhibits cancer stem cell phenotypes in ex vivo models of colorectal liver metastases, and is clinically safe and tolerable in combination with FOLFOX chemotherapy
Source: Cancer Lett. 2015 Aug 10;364(2):135–41. doi: 10.1016/j.canlet.2015.05.005 (PMC4510144; doi:10.1016/j.canlet.2015.05.005)

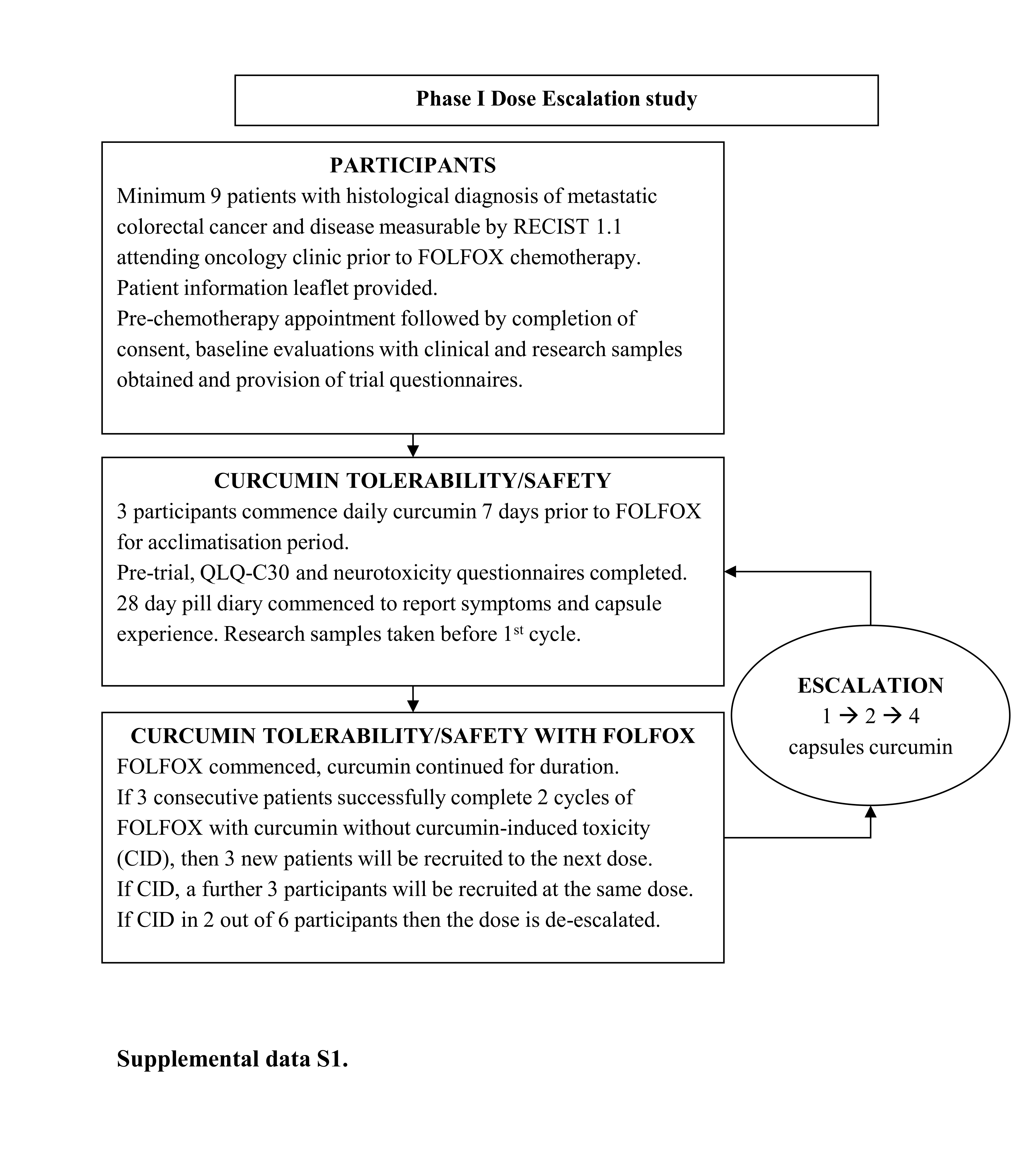

Supplement: Supplemental data S1 — CUFOX Phase I dose escalation trial schema. [file mmc1.zip › mmc1.tif]

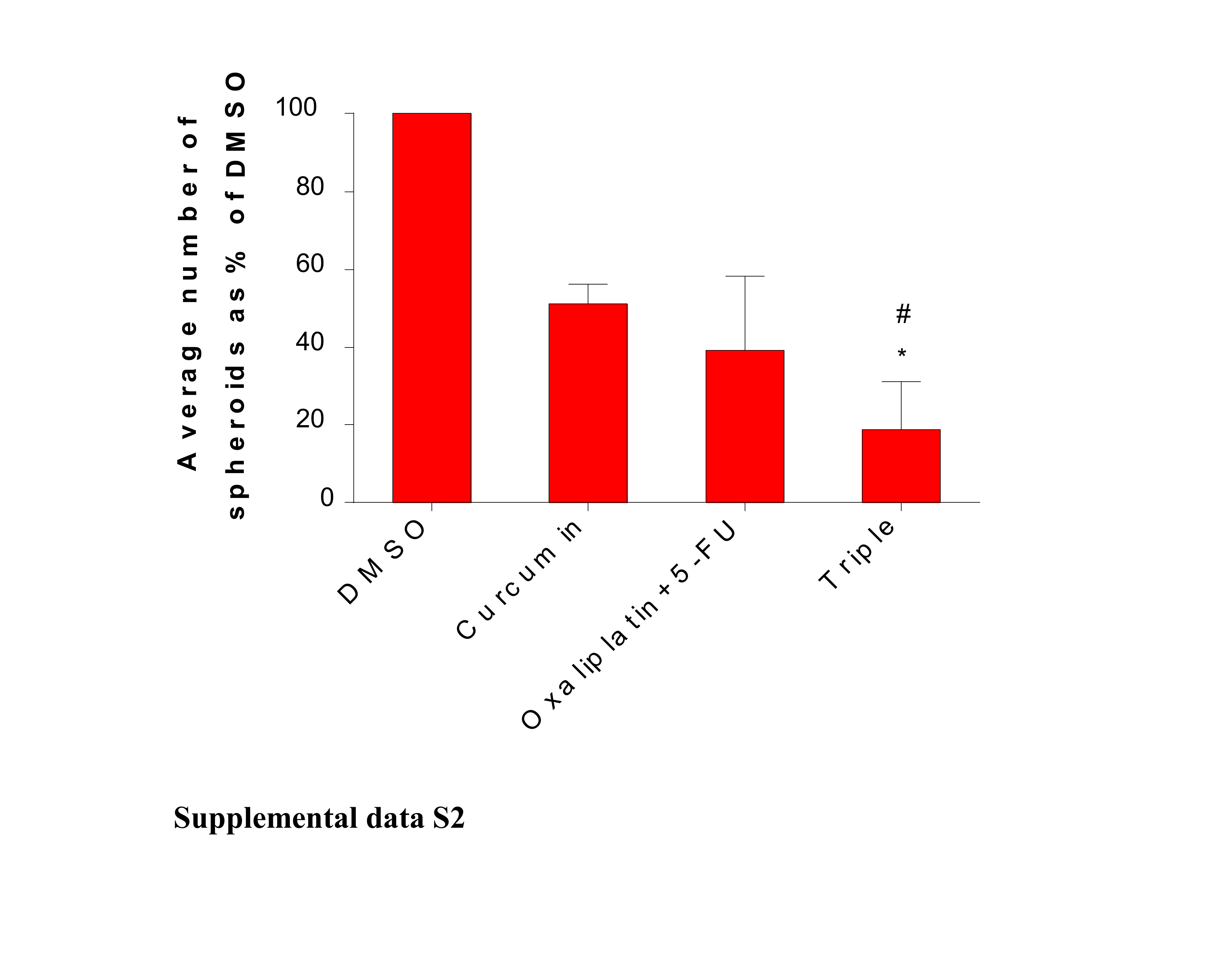

Supplement: Supplemental data S2 — Spheroid number after treatment, represented as a percentage of the DMSO control. N = 5 (each replicate is a different patient sample). ±SEM, * = P ≤ 0.05 compared to DMSO, # = P ≤ 0.05 compared to curcumin alone. [file mmc2.zip › mmc2.tif]

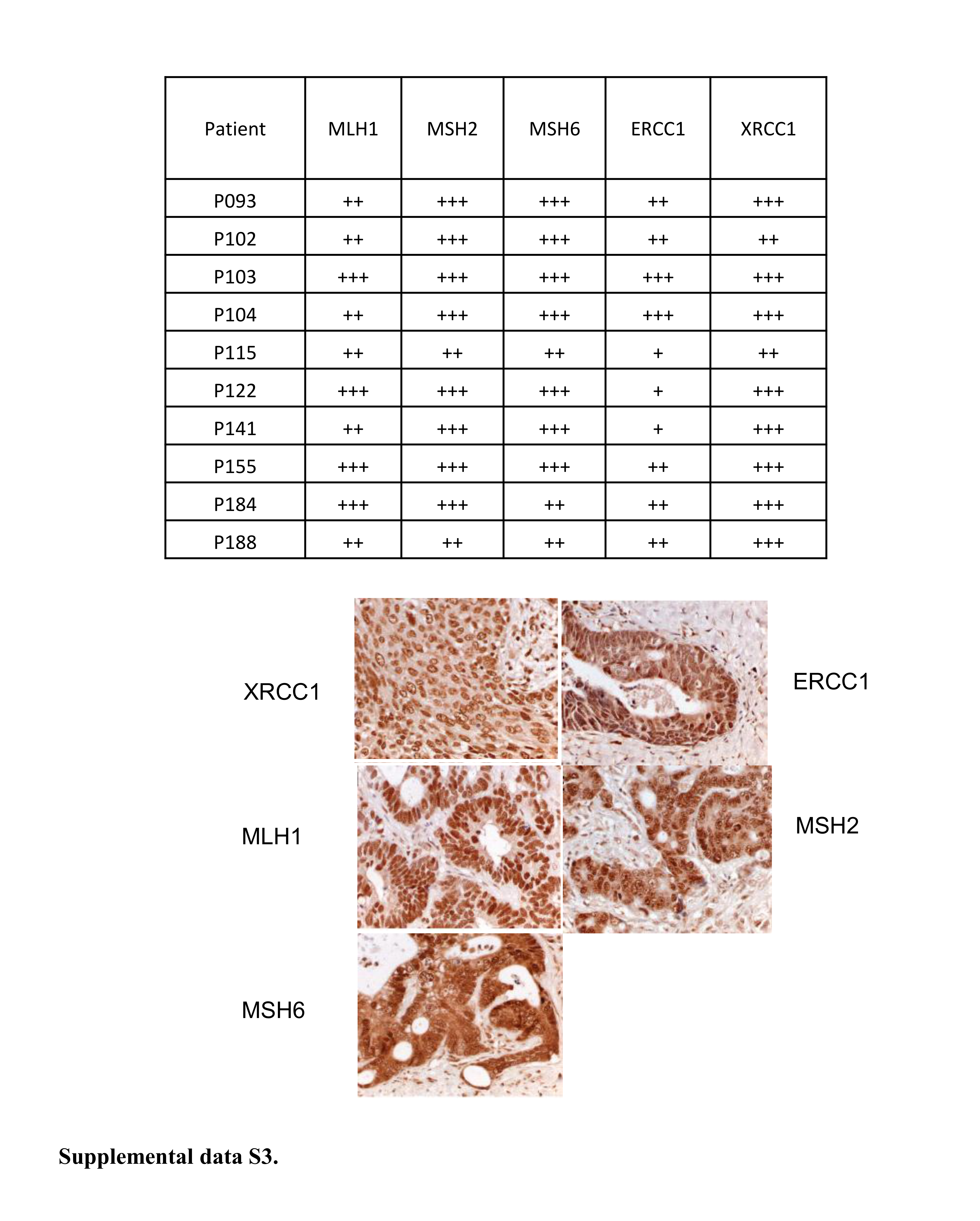

Supplement: Supplemental data S3 — Table shows basal expression of DNA mis-match repair proteins by IHC in the samples used for explant culture, and sample staining patterns. A pathologist assessed the staining for each explant and assigned a score of either +, ++ or +++ with more plus symbols representing a greater amount of expression between samples. No changes to staining patterns were observed following any treatment. [file mmc3.zip › mmc3.tif]

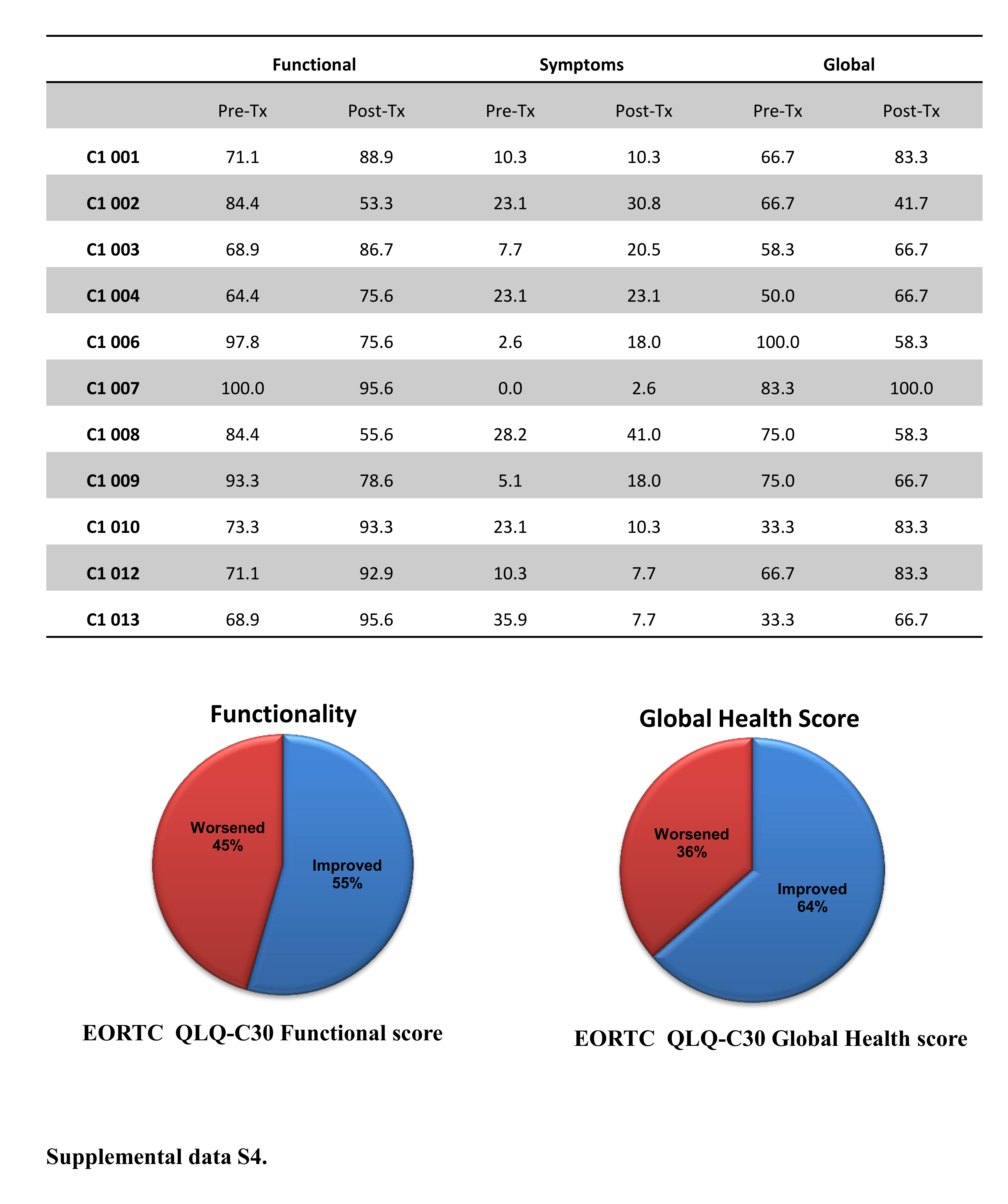

Supplement: Supplemental data S4 — Table shows Global health score and functionality for all participants as assessed using EORTC QLQ30. C1 001, 002, 003 = tier 1, C1004, 006, 007 = tier 2, C1 008, 009, 010, 012, 013 = tier 3. C1 011 for tier 3 = RIP so no post-trial data were obtained. [file mmc4.zip › mmc4.tif]

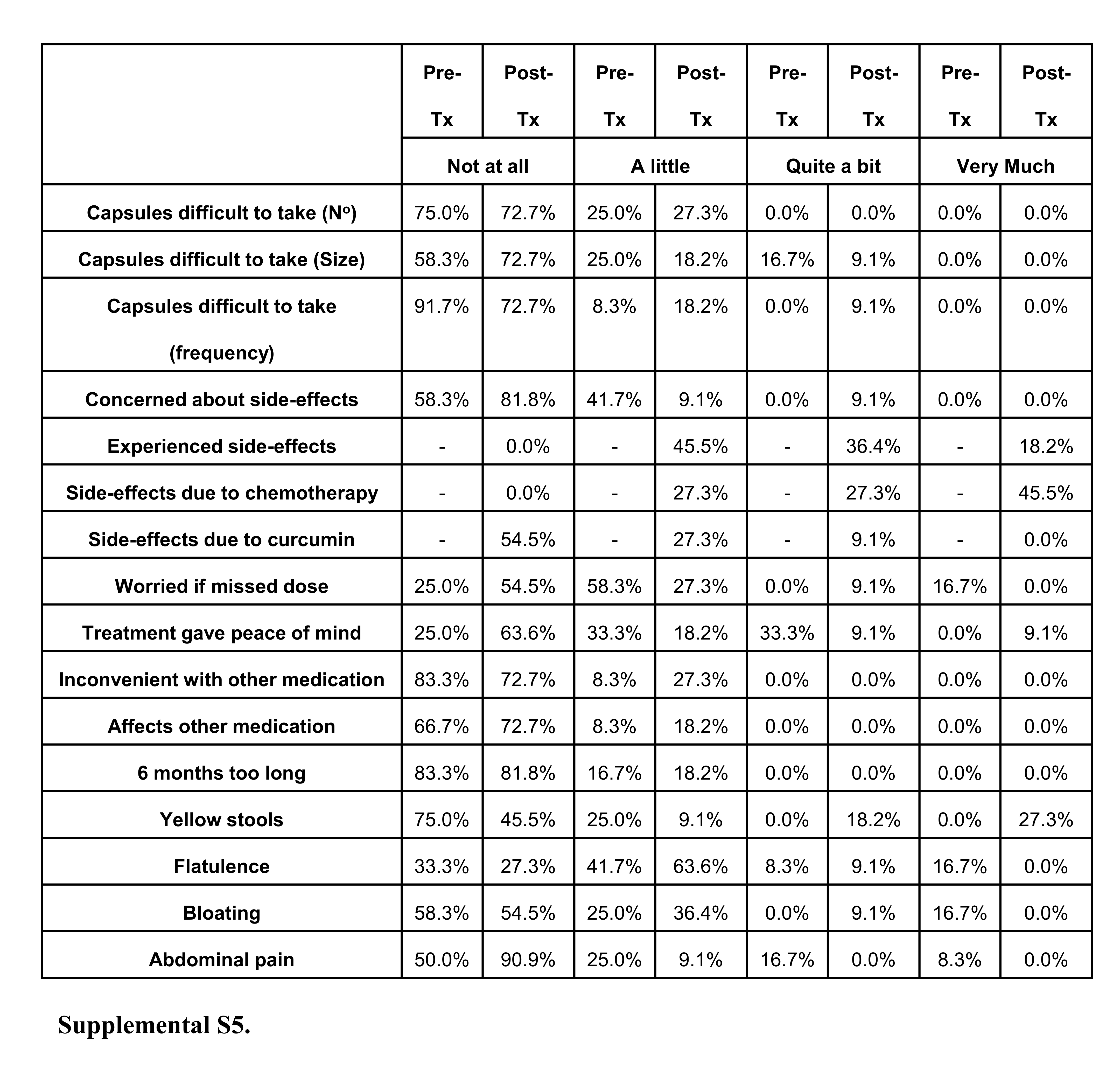

Supplement: Supplemental data S5 — Results of curcumin questionnaires showing responses pre- and post-trial. [file mmc5.zip › mmc5.tif]
